# Immune Cells in Thermogenic Adipose Depots: The Essential but Complex Relationship

**DOI:** 10.3389/fendo.2022.839360

**Published:** 2022-03-14

**Authors:** Marina Agueda-Oyarzabal, Brice Emanuelli

**Affiliations:** Novo Nordisk Foundation Center for Basic Metabolic Research, Faculty of Health and Medical Sciences, University of Copenhagen, Copenhagen, Denmark

**Keywords:** immune cells, batokines, crosstalk, thermogenesis, adipose tissue

## Abstract

Brown adipose tissue (BAT) is a unique organ in mammals capable of dissipating energy in form of heat. Additionally, white adipose tissue (WAT) can undergo browning and perform thermogenesis. In recent years, the research community has aimed to harness thermogenic depot functions for new therapeutic strategies against obesity and the metabolic syndrome; hence a comprehensive understanding of the thermogenic fat microenvironment is essential. Akin to WAT, immune cells also infiltrate and reside within the thermogenic adipose tissues and perform vital functions. As highly plastic organs, adipose depots rely on crucial interplay with these tissue resident cells to conserve their healthy state. Evidence has accumulated to show that different immune cell populations contribute to thermogenic adipose tissue homeostasis and activation through complex communicative networks. Furthermore, new studies have identified -but still not fully characterized further- numerous immune cell populations present in these depots. Here, we review the current knowledge of this emerging field by describing the immune cells that sway the thermogenic adipose depots, and the complex array of communications that influence tissue performance.

## Introduction: Obesity and the Adipose Tissue

Obesity and its underlying metabolic complications are a worldwide concern with significant medical and social implications (1–4). Adipose depots are the primary site of energy storage and also perform vital endocrine functions that regulate and maintain energy homeostasis (5). In mammals, two main types of adipose tissue exist, being different in origin, location, morphology and function (6–9). White adipocytes contain large unilocular lipid droplets that store large amounts of lipids, whereas brown adipocytes contain multilocular small lipid droplets as well as a high number of mitochondria and dissipate energy as heat. Additionally, beige adipocytes originate from a white adipocyte lineage but can undergo browning and acquire brown adipocyte-like features (10). Thus, both brown and beige fat tissues have thermogenic potential and will be referred to as thermogenic adipose tissues (TAT). After its re-discovery in adult humans (11–14), TAT gained considerable therapeutic interest for the treatment of metabolic disease (15, 16). A profound understanding of TAT functions and heterogeneity is required to properly harness its benefits, hence, great focus has been devoted on investigating key cells, such as immune cells, that are critical contributors of TAT health.

## Immune Cells Are Relevant Both in White and Thermogenic Adipose Tissues

TAT not only harbor adipocytes, but stromal cells, nerves, pre-adipocytes and immune cells also reside within these tissues (17–19). Similarly to WAT, where immune cells reside and infiltrate the tissue for healthy expansion and homeostasis (20, 21) or contribute to chronic inflammation during obesity (22–25) immune cells in TAT may support tissue remodeling but might also contribute to alter thermogenic function. As of today, the knowledge about immunity and inflammation in the context of TAT is recent and more limited (26–31).

## Understanding the Roles of Immune Cells in Thermogenic Adipose Depots

The immune system is a complex family of molecules, cells and organs. Immune cells can be classified into myeloid or lymphoid lineages depending on the progenitor cell they arose from (32). Besides, the immune response can be rapid and unspecific (innate response) or slower but more sophisticated (adaptive response), which allows a simple classification of cells based on their origin and their ability to respond to a foreign threat. In absence of danger, immune cells contribute to tissue surveillance and homeostasis (33). In TAT, immune cells are essential not only to maintain tissue homeostasis, but also to help the tissue adapt to external stimuli, such as cold or pathogenic conditions. A summary of all immune cells described to perform important functions in TAT is presented in **Table 1**.

**Table 1:** Overview of the general roles and specific functions of immune cells in thermogenic adipose depots.

Cell	Lineage	Line of defense	Subsets	General functions	Functions in thermogenic adipose depots
**Macrophage**	Myeloid	Innate immunity	M1	Pro-inflammatory. M1 cells exert anti-microbial and anti-tumoral activity. Involved in tissue damage (34).	Promote a pro-inflammatory state. Secretion of pro-inflammatory cytokines. Whitening of BAT and loss of thermogenic potential. Exacerbation of obese conditions (35–37).
			M2a, M2b, M2c, M2d	Anti-inflammatory. M2 cells regulate tissue homeostasis and repair. Phagocytic, angiogenic and immunomodulatory capacity (34).	Promote an anti-inflammatory state. Direct communication with eosinophils through secreted molecules to maintain thermogenic homeostasis. Contribute to browning of WAT possibly through catecholamine or acetylcholine production. A macrophage subset (SAM) impairs thermogenic activation through catecholamine degradation. Discrepancies may arise due to the wide heterogeneity of macrophage populations (38–42).
**Eosinophil**	Myeloid	Innate immunity	–	Involved in defense against parasites and helminths, and allergies (34).	Sustain adaptive thermogenesis by communicating with M2 and ILC2s. Involved in tissue browning and anti-inflammatory state promotion. Genetic loss of eosinophils negatively affects cold responses in TAT (42, 43).
**T cell**	Lymphoid	Adaptive immunity	CD4^+^ T helper (Th)	Th cells orchestrate and modulate adaptive immune responses (34).	Naïve Th cells in TAT differentiate towards Tregs upon cold stimuli. Altered tissue conditions may trigger skewing towards pro-inflammatory subsets (Th1) (44, 45).
			CD4^+^ T regulator (Treg)	Tregs express Foxp3 and are involved in immune suppression and homeostasis (34).	Support and regulate homeostasis in TAT by suppressing inflammatory signals. Treg loss affects cold responses and thermogenic identity. Their alteration through inflammatory signals affects metabolic syndrome (44–46).
			CD8^+^ T cytotoxic (Tc)	Tc cells kill virus infected and cancer cells (34).	Suppression of browning through IFN signaling (47).
**B cell**	Lymphoid	Adaptive immunity	Activated B cell	In charge of antibody production, antigen presentation and production of cytokines (34).	B cells comprise 20-30% of all leukocytes in TAT. Their number increases upon diet-induced obesity, and they negatively modulate IL-10 receptor in beige adipocytes (48, 49).
			Plasma cell		
**Innate lymphoid cell (ILC)**	Lymphoid	Innate immunity	ILC1, ILC2, ILC3	ILCs belong to the lymphoid family but do not express antigen-specific receptors. Thought to be the innate counterparts of Th1, Th2 and Th17 cells (34).	Induction of proliferation of PDGFRα progenitor cells and modulation of M2 macrophages and eosinophils to promote browning. ILC2 populations in TAT are altered during obesity (43, 50, 51).
**γδ T cell**	Lymphoid	Adaptive immunity	Vδ1, Vδ2	γδ T cells express a unique T-cell receptor (γδ TCR) different from conventional δcells (αβ TCR). They have cytotoxic and modulatory capacity (34).	Support Treg function and tissue innervation through IL-17 signaling (52, 53).
**NKT cell**	Lymphoid	Adaptive immunity	Type1, Type2, NKT-like	NKT cells express an invariant TCR α chain and share properties from both NK and T cells. They recognize lipid antigens presented by CD1d (34).	Modulation of Treg homeostasis and function through IL-2 secretion. Induction of FGF-21 production to promote browning (54, 55).
**Monocyte**	Myeloid	Innate immunity	Classical, Non-classical, Intermediate	Monocytes circulate in the blood and infiltrate inflamed tissues to differentiate into macrophages (34).	Support BAT homeostasis by promoting tissue expansion (56).
**Mast cell**	Myeloid	Innate immunity	–	Mast cells contain granules like histamine and play key roles in allergy and anaphylaxis (34).	Mast cells communicate directly with progenitor cells through molecules such as histamine or serotonin, albeit whether this supports or hampers TAT functions remains to be clarified (57–59).

### Macrophages: Patrollers With Wide Functions

Macrophages comprise the most well studied immune cell population in the AT. While the AT literature mostly classifies macrophages into the simple M1/M2 (pro/anti-inflammatory) subsets, macrophages are highly plastic, and many more subsets may be described according to a wide series of markers and functions (60, 61).

Macrophages within WAT and BAT present different profiles (62). BAT macrophages are generally profiled as the M2 subset (63) and have been proposed to support thermogenic functions (27). In addition to their role in BAT, M2 macrophages were also reported to facilitate browning by clearing out dead adipocytes and favoring the recruitment of PDGFRα^+^ progenitor cells after β3-adrenergic receptor activation (64). A later study showed that mice receiving adipose stem cell-derived exosomes induced M2 macrophage activation, favored WAT browning and improved metabolism (65). In another study, the suppression of M2 polarization affected BAT activity and WAT browning in obese mice (35). Conversely, other studies linked the M1 phenotype induction with loss of browning in WAT and impaired BAT thermogenic function (36, 37). Thus, M2 polarization, browning, and BAT functions are positively correlated (38, 39). However, a recent study claimed a subset of M2 macrophages to partially hijack beige progenitor proliferation and browning (66). Overall, the precise link between macrophage alternative activation and thermogenesis still remains to be formally established, and it is important to highlight that a deep categorization of all macrophage populations was missing in these studies, raising the possibility that further distinct subpopulations of macrophages mediate different effects, potentially explaining some discrepancies.

M2 macrophages were initially reported to sustain adaptive thermogenesis through catecholamine production in response to cold (67, 68), bringing a new perspective in the study of TAT modulation through immune cells. However, recent studies disclaimed this fact, as no changes in energy expenditure were found between wild-type, *Ucp1^-/-^* and *Il4ra^-/-^* mice, nor were AT-resident macrophages (ATMs) expressing the catecholamine synthetizing enzyme, tyrosine hydroxylase (69). In fact, a subpopulation of macrophages, the so-called sympathetic neuron-associated macrophages (SAMs), has been suggested to metabolize catecholamines *via* the expression of the norepinephrine (NE) transporter solute carrier family 6 member 2 (SLC6A2) and the degradation enzyme monoamine oxidase A (MAOA) (40). Macrophages were also found to degrade NE *via* NLRP3 inflammasome activation (70). These conflicting results could rise from the fact that macrophages sustain sympathetic innervation (71, 72), and although not directly synthetizing it, they might contribute to the local titers of NE produced by sympathetic neurons present in TAT by directly communicating with them (73, 74). More recently, a study pointed out CX3CR1^+^ SAMs as a potential source of IL-27, which has been shown to contribute to thermogenesis and energy expenditure (75). Besides, a novel study proposed a subpopulation termed cholinergic adipose macrophages (ChAMs) to secrete acetylcholine and regulate thermogenic activation of beige fat (41), adding another layer of complexity to the global contribution of macrophages to sympathetic activation. The sufficiency of M2 macrophages in promoting browning independently of sympathetic neuron involvement was described in a *Fasn* ^-/-^ mouse model (76). Furthermore, macrophages can also express uncoupling protein 1 (UCP1) and aid on beige AT remodeling after cold exposure (77).

Lineage tracing human studies suggested that WAT macrophages could dedifferentiate towards pre-adipocytes and vice versa (78). If applicable to the thermogenic depots, this could become a valuable approach to increase the portion of energy-burning cells within TAT. In other tissues such as the heart, macrophages have been proposed to be essential for mitochondria turnover leading to severe alterations in cardiac function and metabolism when ablated (79). This opens up a new horizon for alternative roles of macrophages in TAT and could lead to new therapeutic approaches.

### Eosinophils and Type 2 Innate Lymphoid Cells: The Type 2 Immunity

Eosinophils and type 2 innate lymphoid cells (ILC2s) are usually present in the mucosas and act during helminthic infections and allergies (80, 81), although their functions are more diverse and vary depending on their location in the body (82). In adipose depots, eosinophils express high levels of Siglec-F and produce IL-4 and IL-13 to induce M2 macrophage polarization (42). Genetic ablation of eosinophils or the IL4/13-IL4Rα-STAT6 pathway has been linked to impaired cold-induced browning as a result of loss of macrophage M2 polarization (67). Therefore, the overall contribution of M2 macrophages to TAT functions and browning may be regulated upstream by the presence of eosinophils and/or IL-4/13 signaling. Type 2 immune signaling could hence be a promising approach for the treatment of metabolic diseases. This strategy was tackled in a study which tried to counteract the HFD-induced eosinopenia by treating mice with helminth antigens (83). Despite increased eosinophils in WAT, the treatment neither did affect eosinophil numbers in BAT nor induced WAT activation or browning. Considering that immune cell profiles vary depending on their environment, the immune response against helminths might have not been enough to induce the same eosinophil profile required for browning *in vivo*. However, advancements in understanding eosinophil plasticity could aid on harnessing type 2 immune responses against metabolic disorders.

Eosinophils infiltrate BAT in response to CCL-11 and IL-5 (43, 83, 84). ILC2s are the major source of IL-5 and their functions are negatively affected during obesity (43). ILC2s sustain adipose eosinophils and macrophages through IL-5 and IL-13 production (43) albeit it has been proposed that ILC2s alone could be sufficient to elicit browning and promote metabolic homeostasis independently from eosinophils (50). Upon activation by IL-33, ILC2s promote PDGFRα^+^ adipocyte precursor proliferation and commitment to the beige lineage *via* IL-4Rα, which overall culminates in increased beige fat mass and improved energy expenditure (51). A new study also revealed an intercoordinated mechanism between ILC2s and eosinophils to enhance sympathetic innervation in WAT (85). Detailed mechanisms by which ILC2s contribute to beige adipogenesis, and metabolism have been described elsewhere (86, 87). Overall, type 2 immune cells are able to directly and indirectly contribute to the positive functions of TAT in energy homeostasis. While the underlying mechanisms are emerging, further research is needed to decipher the complex array of communications between type 2 immune cells and adipocytes.

### Regulatory T Cells and Unconventional T Cell Subsets

Regulatory T cells (Tregs) constitute a CD4^+^ T cell subset that highly expresses the forkhead box P3 (FOXP3) transcription factor and are crucial for the maintenance of self-tolerance and immune homeostasis. AT resident Tregs were only discovered by the end of the last decade (88), however, large-scale research allowed adipose Tregs to be one of the best characterized along all tissues. AT resident Tregs express PPAR-γ (89) as well as a distinct T cell-receptor (TCR) repertoire compared to other Tregs in the lymph node (88). Correspondingly to many other immune cells, Treg characterization and relevance have been more largely described in WAT (90), albeit it is predictable that these cells also play major roles in TAT.

BAT Tregs harbor a specific transcriptomic signature different from splenic Tregs or T conventional (T_conv_) cells in all tissues, including T_conv_ cells in BAT (46). Interestingly, this signature is also different from WAT Tregs, which incites to question whether these cells perform different functions in WAT and TAT. Tregs have been proposed to maintain BAT identity and promote browning (46, 91), and a recent study showed that inflammatory signals mediated by the insulin receptor in visceral WAT and BAT Tregs give rise to detrimental outcomes in the regulation of diet- and age-induced metabolic syndrome (92). Other studies in mouse and humans also showed the presence of resident naïve CD4^+^ T cells that skew towards a regulatory phenotype under certain stimuli (i.e cold), describing them as cold-inducible Tregs (44, 45). Cold-inducible Tregs mainly increase in BAT and beige AT and their depletion automatically affects the thermogenic identity of the tissue. Inversely, UCP1 ablation impairs Treg induction (44). Therefore, each cell type relies on one another to sustain adaptive thermogenesis. Still, whether Treg-adipocyte communication happens directly upon cold exposure or trough complex signaling events that lead to secretion of molecules needs to be investigated.

On the other hand, Tregs resemble the boring parents that shut down the party when it is time to go home. But how are these cells prevented to restrict too harshly? The AT harbors such a wide array of immune cells that it even allows to regulate the regulatory cells. This is the case for invariant natural killer T (iNKT) cells, a singular adipose resident immune cell subset, which tightly modulates the proliferation and suppressor function of adipose Tregs through the secretion of IL-2 (54). Besides, α-galactosylceramide-activated iNKT cells upregulate fibroblast growth factor 21 (FGF-21), which subsequently promotes adipocyte browning (55). Another subset of T cells, the so-called unconventional γδ T cells, has also been shown to support Treg cell homeostasis and tissue innervation through IL-17 signaling (52, 53).

### Other Immune Populations in Thermogenic Adipose Depots

Elucidating all the immune populations in TAT and their contribution to metabolism has become an attractive field of research. More immune populations have been recently described in TAT. Monocytes have been shown to support BAT homeostasis by promoting tissue expansion (56). B cells comprise 20 to 30% of leukocytes in BAT, and their number is further increased upon diet-induced obesity (48). Along with T cells, B cells have been suggested to negatively influence thermogenesis *via* adipocyte IL-10 signaling (49). CD8^+^ T cells have also been suggested to negatively influence beige adipogenesis through IFN-γ secretion in a lymphocyte deficient mouse model (47). Mast cells are also present in TAT and communicate with adipose cells through factors like histamine or serotonin, albeit whether they promote or hamper WAT browning remains to be clarified (57–59). Accordingly, further studies on these populations will be required to sustain the presented findings. More recent studies using single-cell or nuclei RNA sequencing approaches have shown still undescribed immune cell populations, such as neutrophils or dendritic cells, to be present in TAT of mice (56, 76, 93) and humans (93). It is therefore encouraging to expect that the roles of more immune cell populations in thermogenic depots will be unveiled in the coming future. Furthermore, it will be important to not only consider the ability of TAT immune cells to influence tissue identity and function, but also to modulate energy expenditure, following appropriate guidelines (94, 95).

## The Complex and Reciprocal Interplay Between Adipocytes and Immune Cells: Cytokines, Chemokines and Batokines

**Figure 1:**
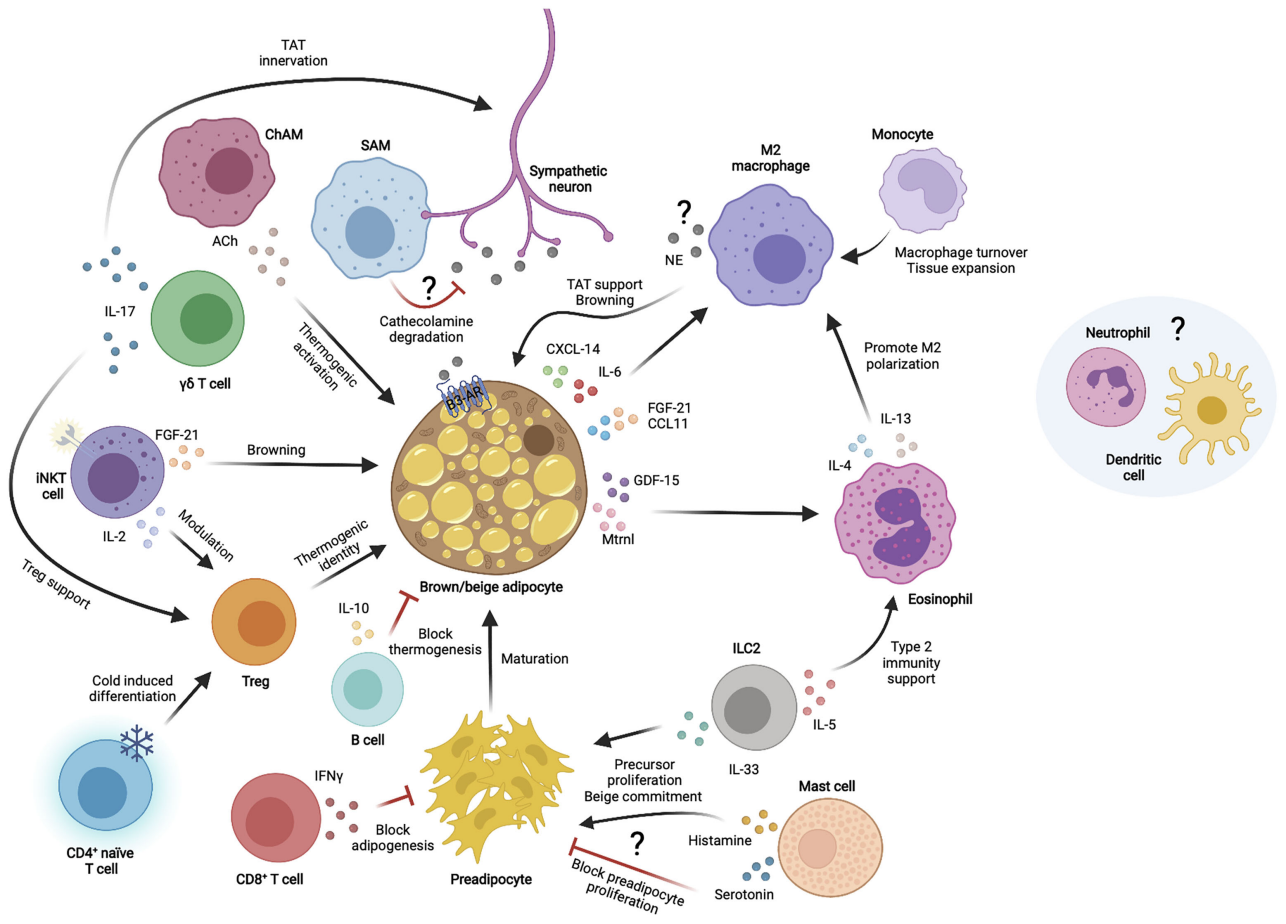
Immune cell interactions with thermogenic adipocytes in the thermogenic adipose tissue microenvironment. Different immune cells communicate with each other and/or thermogenic adipocytes and sympathetic neurons through secreted factors to modulate thermogenic adipose tissue functions. TAT, thermogenic adipose tissue; NE, norepinephrine; Ach, Acetylcholine; IL, interleukin; CXCL, chemokine C-X-C motif ligand; CCL, chemokine C-C motif ligand; FGF-21, fibroblast growth factor 21; GDF-15, growth differentiation factor 15; Mtrnl, meteorin-like; IFNγ, interferon gamma; ILC2, innate lymphoid cell type 2; SAM, sympathetic neuron associated macrophage; ChAM, Cholinergic adipose macrophage; iNKT, invariant natural killer T cell; γδ T cell, gamma-delta T cell; Treg, T regulatory cell; β3-AR, β3 adrenergic receptor.

The TAT environment is full of cells that continuously interact reciprocally to maintain tissue homeostasis and adapt to physiological conditions (**Figure 1**). Some of these intercellular crosstalks include autocrine communication between adipocytes, paracrine communication with immune cells, paracrine communication among different –or newly recruited– immune cells and communications among other cells within the tissue. We have previously outlined some of the cytokines and chemokines by which immune cells communicate among themselves to influence TAT homeostasis and function. Thermogenic adipocytes also secrete many of these factors (in this case called “BAT”okines) to directly communicate with immune cells.

IL-6 was one of the first interleukins found to be secreted by brown adipocytes upon noradrenergic stimulation (96) and was later implied in a direct effect on macrophage polarization and the decline of obesity-associated inflammation in WAT (97). FGF-21 is a circulating factor primarily originating from the liver that performs important metabolic functions, including increasing energy expenditure and browning (98). FGF-21 can be produced from other tissues to mediate local action, including BAT, which is an important source of FGF-21 following cold exposure (99, 100). FGF-21 was later shown to synergize with the chemokine CCL-11 to accumulate M2 macrophages and adipocyte precursor cells to enhance browning (101). Indeed, the type 2 immune response and browning is abolished in absence of FGF-21, whilst only CCL-11 treatment is sufficient to restore these processes, which brings in new key processes coupling immunity and thermogenic fat activation.

A different study reported the meteorin-like (Mtrnl) factor, produced by beige AT upon cold exposure, to communicate with eosinophils to produce IL-4 and promote M2 macrophage skewing, thus enabling an anti-inflammatory environment in BAT and improving glucose tolerance and energy expenditure (102, 103). Another study revealed that CXCL14 is released by brown adipocytes in response to noradrenergic activation to directly induce macrophage recruitment and support thermogenesis in beige WAT (100). Additionally, *in vitro* studies by the same group revealed activated brown adipocytes to directly influence the anti-inflammatory state of macrophages through secreted GDF-15 (104).

The current knowledge on batokines and their targets has been detailly described (105, 106). Proteomics studies have shown many more immune factors such as complement proteins, additional pro- and anti-inflammatory cytokines and chemokines to be part of the brown adipocyte secretome upon its stimulation (107, 108). Furthermore, alternative ways of communication also exist *via* lipid metabolites, extracellular vesicles and micro-RNAs (109–111). So far, the proper characterization of these novel batokines remains vague or inexistent. Some of the identified factors could conserve their chemotactic effects in TAT, as it has been described for CXCL14, and allow the infiltration of certain immune cell subsets to sustain TAT functions and health. Moreover, proteins like complement system factors are known to generally elicit inflammatory responses, making it quite intriguing to decipher whether this function is conserved in the physiological response to cold. Somehow, this would resemble the same first clues arisen around the anti-inflammatory roles of IL-6 in TAT compared to its contrary effects in WAT (112); which endorses the key role of the environment in deciding the fate of immunological factors.

Overall, the mechanisms by which adipocytes communicate with the immune environment are just starting to be uncovered. Unraveling these complex interactive networks remains a challenge and still requires profound investigation.

## Immune Cell Recruitment to TAT: Infiltration Is Not Inflammation

It is now evident that TAT secretes factors upon activation that target immune cells. Based on this, one would think that the primary response to cold is to produce an inflammatory state in the tissue, which would affect TAT function. However, based on the immune cells that harbor and infiltrate TAT, one realizes that it is not the cells *per se* but their state that impacts tissue condition and performance. Most of the immune cells present in TAT show an anti-inflammatory phenotype, and the physiological response to cold seems to further impulse this state. The secretory profile of activated brown adipocytes converges with this idea, as immunosuppressor proteins such as IL-10 or the complement factor H are found in these data sets (107, 108). Furthermore, the shift towards pro-inflammatory states and signals are associated with the whitening of BAT, decreased energy expenditure and altered metabolism both in mouse and humans (35, 74, 113–115). Consequently, it is tempting to think that not only does TAT contribute to metabolic homeostasis by burning excess fat mass, but also by generating an overall anti-inflammatory environment that could even be spread in an endocrine manner to the white depots, thus helping to restrain obesity induced chronic inflammation (116).

## Closing Remarks and Further Perspectives

The improved metabolic profile of animals and patients upon TAT activation turns this energy burning depot into an appealing alternative therapy ameliorating metabolic health. However, TAT is a heterogeneous tissue and many cells beyond brown adipocytes exert indispensable roles. Many immune cells contribute and tightly regulate metabolic processes that allow the normal function of the tissue. Akin, signal alterations consequence of the obese profile directly shift TAT immune cell behavior subsequently influencing thermogenic adipocyte responses. This tight link between immune cells and thermogenic adipocytes could be harnessed to boost thermogenic functions and improve the overall complications associated with obesity. Whether this could be approached through secreted molecules or by directly targeting specific cell populations will need deeper understanding. Despite being a rather novel field of research, promising animal studies that alter immune components in TAT provide reasonable hope that immune modulation of TAT may become an attractive therapeutic strategy to positively impact human metabolic health.

## Author Contributions

MA-O wrote the manuscript. BE contributed to manuscript structure, discussion of ideas, and revision. All authors contributed to the article and approved the submitted version.

## Funding

This work was supported by the Novo Nordisk Foundation Center for Basic Metabolic Research, an independent research center at the University of Copenhagen partially funded by an unrestricted donation from the Novo Nordisk Foundation (NNF18CC0034900). MA-O is supported by the Copenhagen Bioscience PhD program and the Novo Nordisk Foundation (NNF19SA0035436).

## Conflict of Interest

The authors declare that the research was conducted in the absence of any commercial or financial relationships that could be construed as a potential conflict of interest.

## Publisher’s Note

All claims expressed in this article are solely those of the authors and do not necessarily represent those of their affiliated organizations, or those of the publisher, the editors and the reviewers. Any product that may be evaluated in this article, or claim that may be made by its manufacturer, is not guaranteed or endorsed by the publisher.

## References

[1] Ng M Fleming T Robinson M Thomson B Graetz N Margono C. Global, Regional, and National Prevalence of Overweight and Obesity in Children and Adults During 1980-2013: A Systematic Analysis for the Global Burden of Disease Study 2013. Lancet (2014) 384:766–81. doi: 10.1016/S0140-6736(14)60460-8 PMC4624264 24880830

[2] Must A Spadano J Coakley EH Field AE Colditz G Dietz WH. The Disease Burden Associated With Overweight and Obesity. J Am Med Assoc (1999) 282:1523–9. doi: 10.1001/jama.282.16.1523 10546691

[3] Stein CJ Colditz GA. The Epidemic of Obesity. J Clin Endocrinol Metab (2004) 89:2522–5. doi: 10.1210/jc.2004-0288 15181019

[4] Malik VS Willett WC Hu FB. Global Obesity: Trends, Risk Factors and Policy Implications. Nat Rev Endocrinol (2013) 9:13–27. doi: 10.1038/nrendo.2012.199 23165161

[5] Funcke JB Scherer PE. Beyond Adiponectin and Leptin: Adipose Tissue-Derived Mediators of Inter-Organ Communication. J Lipid Res (2019) 60:1648–97. doi: 10.1194/jlr.R094060 PMC6795086 31209153

[6] Rosen ED Spiegelman BM. What We Talk About When We Talk About Fat. Cell (2014) 156:20–44. doi: 10.1016/j.cell.2013.12.012 24439368 PMC3934003

[7] Tchkonia T Thomou T Zhu Y Karagiannides I Pothoulakis C Jensen MD. Mechanisms and Metabolic Implications of Regional Differences Among Fat Depots. Cell Metab (2013) 17:644–56. doi: 10.1016/j.cmet.2013.03.008 PMC3942783 23583168

[8] Zwick RK Guerrero-Juarez CF Horsley V Plikus MV. Anatomical, Physiological, and Functional Diversity of Adipose Tissue. Cell Metab (2018) 27:68–83. doi: 10.1016/j.cmet.2017.12.002 29320711 PMC6050204

[9] Peirce V Carobbio S Vidal-Puig A. The Different Shades of Fat. Nature (2014) 510:76–83. doi: 10.1038/nature13477 24899307

[10] Herz CT Kiefer FW. Adipose Tissue Browning in Mice and Humans. J Endocrinol (2019) 241:R97–R109. doi: 10.1530/JOE-18-0598 31144796

[11] Nedergaard J Bengtsson T Cannon B. Unexpected Evidence for Active Brown Adipose Tissue in Adult Humans. Am J Physiol - Endocrinol Metab (2007) 293:444–52. doi: 10.1152/ajpendo.00691.2006 17473055

[12] Virtanen KA Lidell ME Orava J Heglind M Westergren R Niemi T. Functional Brown Adipose Tissue in Healthy Adults. N Engl J Med (2009) 360:1518–25. doi: 10.1056/NEJMOA0808949/SUPPL_FILE/NEJM_VIRTANEN_1518SA1.PDF 19357407

[13] van Marken Lichtenbelt WD Vanhommerig JW Smulders NM Drossaerts JMAFL Kemerink GJ Bouvy ND. Cold-Activated Brown Adipose Tissue in Healthy Men. N Engl J Med (2009) 360:1500–8. doi: 10.1056/NEJMOA0808718 19357405

[14] Cypess AM Lehman S Williams G Tal I Rodman D Goldfine AB. Identification and Importance of Brown Adipose Tissue in Adult Humans. N Engl J Med (2009) 360:1509–17. doi: 10.1056/NEJMOA0810780/SUPPL_FILE/NEJM_CYPESS_1509SA1.PDF PMC2859951 19357406

[15] Herz CT Kulterer OC Prager M Schmöltzer C Langer FB Prager G. Active Brown Adipose Tissue Is Associated With a Healthier Metabolic Phenotype in Obesity. Diabetes (2022) 71:93–103. doi: 10.2337/DB21-0475 34957487

[16] Becher T Palanisamy S Kramer DJ Eljalby M Marx SJ Wibmer AG. Brown Adipose Tissue Is Associated With Cardiometabolic Health. Nat Med (2021) 27:58–65. doi: 10.1038/S41591-020-1126-7 33398160 PMC8461455

[17] Troike KM Lee KY List EO Berryman DE. The Complexity of Adipose Tissue. In: Textbook of Energy Balance, Neuropeptide Hormones, and Neuroendocrine Function. Cham, Switzerland: Springer, Cham (2018). doi: 10.1007/978-3-319-89506-2_8

[18] Sun W Modica S Dong H Wolfrum C. Plasticity and Heterogeneity of Thermogenic Adipose Tissue. Nat Metab (2021) 3:751–61. doi: 10.1038/S42255-021-00417-4 34158657

[19] Knights AJ Wu J Tseng YH. The Heating Microenvironment: Intercellular Cross Talk Within Thermogenic Adipose Tissue. Diabetes (2020) 69:1599–604. doi: 10.2337/DB20-0303 PMC7372068 32690661

[20] Shamsi F Wang CH Tseng YH. The Evolving View of Thermogenic Adipocytes - Ontogeny, Niche and Function. Nat Rev Endocrinol (2021) 17:726–44. doi: 10.1038/S41574-021-00562-6 PMC8814904 34625737

[21] Cox AR Chernis N Masschelin PM Hartig SM. Immune Cells Gate White Adipose Tissue Expansion. Endocrinology (2019) 160:1645–58. doi: 10.1210/en.2019-00266 PMC6591013 31107528

[22] Kane H Lynch L. Innate Immune Control of Adipose Tissue Homeostasis. Trends Immunol (2019) 40:857–72. doi: 10.1016/j.it.2019.07.006 31399336

[23] Monteiro R Azevedo I. Chronic Inflammation in Obesity and the Metabolic Syndrome. Mediators Inflamm (2010) 2010. doi: 10.1155/2010/289645 PMC2913796 20706689

[24] Han JM Levings MK. Adipose Inflammation Immune Regulation in Obesity-Associated. J Immunol (2013) 191:527–32. doi: 10.4049/jimmunol.1301035 23825387

[25] Liu R Nikolajczyk BS. Tissue Immune Cells Fuel Obesity-Associated Inflammation in Adipose Tissue and Beyond. Front Immunol (2019) 10. doi: 10.3389/fimmu.2019.01587 PMC6653202 31379820

[26] Chait A den Hartigh LJ. Adipose Tissue Distribution, Inflammation and Its Metabolic Consequences, Including Diabetes and Cardiovascular Disease. Front Cardiovasc Med (2020) 7. doi: 10.3389/fcvm.2020.00022 PMC7052117 32158768

[27] Villarroya F Cereijo R Villarroya J Gavaldà-Navarro A Giralt M. Toward an Understanding of How Immune Cells Control Brown and Beige Adipobiology. Cell Metab (2018) 27:954–61. doi: 10.1016/j.cmet.2018.04.006 29719233

[28] Villarroya F Cereijo R Gavaldà-Navarro A Villarroya J Giralt M. Inflammation of Brown/Beige Adipose Tissues in Obesity and Metabolic Disease. J Internal Med (2018) 284:492–504. doi: 10.1111/joim.12803 29923291

[29] Omran F Christian M. Inflammatory Signaling and Brown Fat Activity. Front Endocrinol (2020) 11:156. doi: 10.3389/fendo.2020.00156 PMC7105810 32265845

[30] Reilly SM Saltiel AR. Adapting to Obesity With Adipose Tissue Inflammation. Nat Rev Endocrinol (2017) 13:633–43. doi: 10.1038/nrendo.2017.90 28799554

[31] Lackey DE Olefsky JM. Regulation of Metabolism by the Innate Immune System. Nat Rev Endocrinol (2015) 12:15–28. doi: 10.1038/nrendo.2015.189 26553134

[32] Kondo M. Lymphoid and Myeloid Lineage Commitment in Multipotent Hematopoietic Progenitors. Immunol Rev (2010) 238:37–46. doi: 10.1111/J.1600-065X.2010.00963.X 20969583 PMC2975965

[33] Sattler S. The Role of the Immune System Beyond the Fight Against Infection. In: Advances in Experimental Medicine and Biology. Springer New York LLC : Springer International. (2017). p. 3–14. doi: 10.1007/978-3-319-57613-8_1 28667551

[34] Abbas AK Lichtman AH Pillai S. Cellular and Molecular Immunology (2021). Available at: https://www.elsevier.com/books/cellular-and-molecular-immunology/abbas/978-0-323-75748-5.

[35] Shan B Wang X Wu Y Xu C Xia Z Dai J. The Metabolic ER Stress Sensor IRE1α Suppresses Alternative Activation of Macrophages and Impairs Energy Expenditure in Obesity. Nat Immunol (2017) 18:519–29. doi: 10.1038/ni.3709 28346409

[36] Sakamoto T Takahashi N Sawaragi Y Naknukool S Yu R Goto T. Inflammation Induced by RAW Macrophages Suppresses UCP1 mRNA Induction *via* ERK Activation in 10T1/2 Adipocytes. Am J Physiol - Cell Physiol (2013) 304:C729–38. doi: 10.1152/ajpcell.00312.2012 PMC3625802 23302779

[37] Gonzalez-Hurtado E Lee J Choi J Wolfgang MJ. Fatty Acid Oxidation Is Required for Active and Quiescent Brown Adipose Tissue Maintenance and Thermogenic Programing. Mol Metab (2018) 7:45–56. doi: 10.1016/j.molmet.2017.11.004 29175051 PMC5784326

[38] Jeon EJ Kim DY Lee NH Choi HE Cheon HG. Telmisartan Induces Browning of Fully Differentiated White Adipocytes *via* M2 Macrophage Polarization. Sci Rep (2019) 10:1236. doi: 10.1038/s41598-018-38399-1 PMC6362091 30718686

[39] van den Berg SM van Dam AD Rensen PCN de Winther MPJ Lutgens E. Immune Modulation of Brown(Ing) Adipose Tissue in Obesity. Endocr Rev (2017) 38:46–68. doi: 10.1210/er.2016-1066 27849358

[40] Pirzgalska RM Seixas E Seidman JS Link VM Sánchez NM Mahú I. Sympathetic Neuron-Associated Macrophages Contribute to Obesity by Importing and Metabolizing Norepinephrine. Nat Med (2017) 23:1309–28. doi: 10.1038/nm.4422 PMC7104364 29035364

[41] Knights AJ Liu S Ma Y Nudell VS Perkey E Sorensen MJ. Acetylcholine-Synthesizing Macrophages in Subcutaneous Fat Are Regulated by β 2 -Adrenergic Signaling. EMBO J (2021) 40:e106061. doi: 10.15252/EMBJ.2020106061 34459015 PMC8672283

[42] Wu D Molofsky AB Liang HE Ricardo-Gonzalez RR Jouihan HA Bando JK. Eosinophils Sustain Adipose Alternatively Activated Macrophages Associated With Glucose Homeostasis. Science (2011) 332:243–7. doi: 10.1126/science.1201475 PMC3144160 21436399

[43] Molofsky AB Nussbaum JC Liang HE Dyken SJV Cheng LE Mohapatra A. Innate Lymphoid Type 2 Cells Sustain Visceral Adipose Tissue Eosinophils and Alternatively Activated Macrophages. J Exp Med (2013) 210:535–49. doi: 10.1084/jem.20121964 PMC3600903 23420878

[44] Kälin S Becker M Ott VB Serr I Hosp F Mollah MMH. A Stat6/Pten Axis Links Regulatory T Cells With Adipose Tissue Function. Cell Metab (2017) 26:475–92.e7. doi: 10.1016/j.cmet.2017.08.008 28877454 PMC5627977

[45] Becker M Serr I Salb VK Ott VB Mengel L Blüher M. Short-Term Cold Exposure Supports Human Treg Induction *In Vivo* . Mol Metab (2019) 28:73–82. doi: 10.1016/j.molmet.2019.08.002 31427184 PMC6822223

[46] Medrikova D Sijmonsma TP Sowodniok K Richards DM Delacher M Sticht C. Brown Adipose Tissue Harbors a Distinct Sub-Population of Regulatory T Cells. PloS One (2015) 10:1–13. doi: 10.1371/journal.pone.0118534 PMC4340926 25714366

[47] Moysidou M Karaliota S Kodela E Salagianni M Koutmani Y Katsouda A. CD8+ T Cells in Beige Adipogenesis and Energy Homeostasis. JCI Insight (2018) 3:1–17. doi: 10.1172/jci.insight.95456 PMC5922290 29515042

[48] Peterson KR Flaherty DK Hasty AH. Obesity Alters B Cell and Macrophage Populations in Brown Adipose Tissue. Obesity (2017) 25:1881–4. doi: 10.1002/oby.21982 PMC5679082 28922564

[49] Rajbhandari P Arneson D Hart SK Ahn IS Diamante G Santos LC. Single Cell Analysis Reveals Immune Cell-Adipocyte Crosstalk Regulating the Transcription of Thermogenic Adipocytes. eLife (2019) 8:e49501. doi: 10.7554/eLife.49501 31644425 PMC6837845

[50] Brestoff JR Kim BS Saenz SA Stine RR Monticelli LA Sonnenberg GF. Group 2 Innate Lymphoid Cells Promote Beiging of White Adipose Tissue and Limit Obesity. Nature (2015) 519:242–6. doi: 10.1038/nature14115 PMC4447235 25533952

[51] Lee MW Odegaard JI Mukundan L Qiu Y Molofsky AB Nussbaum JC. Activated Type 2 Innate Lymphoid Cells Regulate Beige Fat Biogenesis. Cell (2015) 160:74–87. doi: 10.1016/j.cell.2014.12.011 25543153 PMC4297518

[52] Kohlgruber AC Gal-Oz ST Lamarche NM Shimazaki M Duquette D Nguyen HN. γδ T Cells Producing Interleukin-17A Regulate Adipose Regulatory T Cell Homeostasis and Thermogenesis/631/250/256/631/250/2504 Article. Nat Immunol (2018) 19:464–74. doi: 10.1038/s41590-018-0094-2 PMC8299914 29670241

[53] Hu B Jin C Zeng X Resch JM Jedrychowski MP Yang Z. γδ T Cells and Adipocyte IL-17RC Control Fat Innervation and Thermogenesis. Nature (2020) 578:610–4. doi: 10.1038/s41586-020-2028-z PMC7055484 32076265

[54] Lynch L Michelet X Zhang S Brennan PJ Moseman A Lester C. Regulatory iNKT Cells Lack Expression of the Transcription Factor PLZF and Control the Homeostasis of T Reg Cells and Macrophages in Adipose Tissue. Nat Immunol (2015) 16:85–95. doi: 10.1038/ni.3047 25436972 PMC4343194

[55] Lynch L Hogan AE Duquette D Lester C Banks A LeClair K. iNKT Cells Induce FGF21 for Thermogenesis and Are Required for Maximal Weight Loss in GLP1 Therapy. Cell Metab (2016) 24:510–9. doi: 10.1016/j.cmet.2016.08.003 PMC5061124 27593966

[56] Gallerand A Stunault MI Merlin J Luehmann HP Sultan DH Firulyova MM. Brown Adipose Tissue Monocytes Support Tissue Expansion. Nat Commun (2021) 12:5255. doi: 10.1038/S41467-021-25616-1 34489438 PMC8421389

[57] Finlin BS Zhu B Confides AL Westgate PM Harfmann BD Dupont-Versteegden EE. Mast Cells Promote Seasonal White Adipose Beiging in Humans. Diabetes (2017) 66:1237–46. doi: 10.2337/DB16-1057 PMC5399616 28250021

[58] Finlin BS Confides AL Zhu B Boulanger MC Memetimin H Taylor KW. Adipose Tissue Mast Cells Promote Human Adipose Beiging in Response to Cold. Sci Rep (2019) 9:1–10. doi: 10.1038/s41598-019-45136-9 31209239 PMC6572779

[59] Zhang X Wang X Yin H Zhang L Feng A Zhang QX. Functional Inactivation of Mast Cells Enhances Subcutaneous Adipose Tissue Browning in Mice. Cell Rep (2019) 28:792–803.e4. doi: 10.1016/J.CELREP.2019.06.044 31315055 PMC6662660

[60] Martinez FO Gordon S. The M1 and M2 Paradigm of Macrophage Activation: Time for Reassessment. F1000Prime Rep (2014) 6–13. doi: 10.12703/P6-13 24669294 PMC3944738

[61] Chávez-Galán L Olleros ML Vesin D Garcia I. Much More Than M1 and M2 Macrophages, There are Also CD169+ and TCR+ Macrophages. Front Immunol (2015) 6. doi: 10.3389/fimmu.2015.00263 PMC4443739 26074923

[62] Teresa Ortega M Xie L Mora S Chapes SK. Evaluation of Macrophage Plasticity in Brown and White Adipose Tissue. Cell Immunol (2011) 271:124–33. doi: 10.1016/j.cellimm.2011.06.012 PMC3168070 21757190

[63] Thomas D Apovian C. Macrophage Functions in Lean and Obese Adipose Tissue. Metabol: Clin Exp (2017) 72:120–43. doi: 10.1016/j.metabol.2017.04.005 PMC5516622 28641779

[64] Lee Y-H Petkova AP Granneman JG. Identification of an Adipogenic Niche for Adipose Tissue Remodeling and Restoration. Cell Metab (2013) 18:355–67. doi: 10.1016/J.CMET.2013.08.003 PMC4185305 24011071

[65] Zhao H Shang Q Pan Z Bai Y Li Z Zhang H. Exosomes From Adipose-Derived Stem Cells Attenuate Adipose Inflammation and Obesity Through Polarizing M2 Macrophages and Beiging in White Adipose Tissue. Diabetes (2018) 67:235–47. doi: 10.2337/db17-0356 29133512

[66] Igarashi Y Nawaz A Kado T Bilal M Kuwano T Yamamoto S. Partial Depletion of CD206-Positive M2-Like Macrophages Induces Proliferation of Beige Progenitors and Enhances Browning After Cold Stimulation. Sci Rep (2018) 8:14567 doi: 10.1038/s41598-018-32803-6 30275453 PMC6167387

[67] Qiu Y Nguyen KD Odegaard JI Cui X Tian X Locksley RM. Eosinophils and Type 2 Cytokine Signaling in Macrophages Orchestrate Development of Functional Beige Fat. Cell (2014) 157:1292–308. doi: 10.1016/j.cell.2014.03.066 PMC4129510 24906148

[68] Nguyen K Qiu Y Cui X. Alternatively Activated Macrophages Produce Catecholamines to Sustain Adaptive Thermogenesis. Nature (2011) 480:104–108. doi: 10.1038/nature10653 22101429 PMC3371761

[69] Fischer K Ruiz HH Jhun K Finan B Oberlin DJ van der Heide V. Alternatively Activated Macrophages Do Not Synthesize Catecholamines or Contribute to Adipose Tissue Adaptive Thermogenesis. Nat Med (2017) 23:623–30. doi: 10.1038/NM.4316 PMC5420449 28414329

[70] Camell CD Sander J Spadaro O Lee A Nguyen KY Wing A. Inflammasome-Driven Catecholamine Catabolism in Macrophages Blunts Lipolysis During Ageing. Nature (2017) 550:119–23. doi: 10.1038/nature24022 PMC5718149 28953873

[71] Blaszkiewicz M Wood E Koizar S Willows J Anderson R Tseng Y-H. The Involvement of Neuroimmune Cells in Adipose Innervation. Mol Med (2020) 26:1–19. doi: 10.1186/S10020-020-00254-3 PMC7727151 33297933

[72] Wang YN Tang Y He Z Ma H Wang L Liu Y. Slit3 Secreted From M2-Like Macrophages Increases Sympathetic Activity and Thermogenesis in Adipose Tissue. Nat Metab (2021) 3:1536–51. doi: 10.1038/S42255-021-00482-9 34782792

[73] Wolf Y Boura-Halfon S Cortese N Haimon Z Sar Shalom H Kuperman Y. Brown-Adipose-Tissue Macrophages Control Tissue Innervation and Homeostatic Energy Expenditure. Nat Immunol (2017) 18:665–74. doi: 10.1038/ni.3746 PMC5438596 28459435

[74] Rached MT Millership SJ Pedroni SMA Choudhury AI Costa ASH Hardy DG. Deletion of Myeloid IRS2 Enhances Adipose Tissue Sympathetic Nerve Function and Limits Obesity. Mol Metab (2019) 20:38–50. doi: 10.1016/j.molmet.2018.11.010 30553769 PMC6358539

[75] Wang Q Li D Cao G Shi Q Zhu J Zhang M. IL-27 Signalling Promotes Adipocyte Thermogenesis and Energy Expenditure. Nature (2021) 600:314–8. doi: 10.1038/S41586-021-04127-5 34819664

[76] Henriques F Bedard AH Guilherme A Kelly M Chi J Zhang P. Single-Cell RNA Profiling Reveals Adipocyte to Macrophage Signaling Sufficient to Enhance Thermogenesis. Cell Rep (2020) 32. doi: 10.1016/j.celrep.2020.107998 PMC7433376 32755590

[77] Finlin BS Memetimin H Confides AL Zhu B Westgate PM Dupont-Versteegden EE. Macrophages Expressing Uncoupling Protein 1 Increase in Adipose Tissue in Response to Cold in Humans. Sci Rep (123AD) 11:23598. doi: 10.1038/s41598-021-03014-3 34880313 PMC8655049

[78] Chazenbalk G Bertolotto C Heneidi S Jumabay M Trivax B Aronowitz J. Novel Pathway of Adipogenesis Through Cross-Talk Between Adipose Tissue Macrophages, Adipose Stem Cells and Adipocytes: Evidence of Cell Plasticity. PloS One (2011) 6:e17834. doi: 10.1371/journal.pone.0017834 21483855 PMC3069035

[79] Nicolás-Ávila JA Lechuga-Vieco AV Esteban-Martínez L Sánchez-Díaz M Díaz-García E Santiago DJ. A Network of Macrophages Supports Mitochondrial Homeostasis in the Heart. Cell (2020) 183:94–109.e23. doi: 10.1016/j.cell.2020.08.031 32937105

[80] Bochner BS. The Eosinophil: For Better or Worse, in Sickness and in Health. Ann Allergy Asthma Immunol (2018) 121:150–5. doi: 10.1016/j.anai.2018.02.031 PMC6087501 29499369

[81] Bartemes KR Kita H. Roles of Innate Lymphoid Cells (ILCs) in Allergic Diseases: The 10-Year Anniversary for ILC2s. J Allergy Clin Immunol (2021) 147:1531–47. doi: 10.1016/J.JACI.2021.03.015 PMC8114584 33965091

[82] Marichal T Mesnil C Bureau F. Homeostatic Eosinophils: Characteristics and Functions. Front Med (2017) 4. doi: 10.3389/fmed.2017.00101 PMC5504169 28744457

[83] van den Berg SM van Dam AD Kusters PJH Beckers L Den Toom M van der Velden S. Helminth Antigens Counteract a Rapid High-Fat Diet-Induced Decrease in Adipose Tissue Eosinophils. J Mol Endocrinol (2017) 59:245–55. doi: 10.1530/JME-17-0112 28694301

[84] Rozenberg P Reichman H Zab-Bar I Itan M Pasmanik-Chor M Bouffi C. CD300f:IL-5 Cross-Talk Inhibits Adipose Tissue Eosinophil Homing and Subsequent IL-4 Production. Sci Rep (2017) 7:1–15. doi: 10.1038/s41598-017-06397-4 28725048 PMC5517555

[85] Meng X Qian X Ding X Wang W Yin X Zhuang G. Eosinophils Regulate Intra-Adipose Axonal Plasticity. Proc Natl Acad Sci (2022) 119:e2112281119. doi: 10.1073/PNAS.2112281119 35042776 PMC8784130

[86] Flach M Diefenbach A. Adipose Tissue: ILC2 Crank Up the Heat. Cell Metab (2015) 21:152–3. doi: 10.1016/j.cmet.2015.01.015 25651167

[87] Bénézech C Jackson-Jones LH. ILC2 Orchestration of Local Immune Function in Adipose Tissue. Front Immunol (2019) 10. doi: 10.3389/fimmu.2019.00171 PMC6374325 30792718

[88] Feuerer M Herrero L Cipolletta D Naaz A Wong J Nayer A. Lean, But Not Obese, Fat Is Enriched for a Unique Population of Regulatory T Cells That Affect Metabolic Parameters. Nat Med (2009) 15:930–9. doi: 10.1038/nm.2002 PMC3115752 19633656

[89] Cipolletta D Feuerer M Li A Kamei N Lee J Shoelson SE. PPAR-γ is a Major Driver of the Accumulation and Phenotype of Adipose Tissue T Reg Cells. Nature (2012) 486:549–53. doi: 10.1038/nature11132 PMC3387339 22722857

[90] Zeng Q Sun X Xiao L Xie Z Bettini M Deng T. A Unique Population: Adipose-Resident Regulatory T Cells. Front Immunol (2018) 9. doi: 10.3389/fimmu.2018.02075 PMC6172295 30323806

[91] Fang W Deng Z Benadjaoud F Yang D Yang C Shi GP. Regulatory T Cells Promote Adipocyte Beiging in Subcutaneous Adipose Tissue. FASEB J: Off Publ Fed Am Soc Exp Biol (2020) 34:9755–70. doi: 10.1096/FJ.201902518R 32510702

[92] Wu D Wong CK Han JM Orban PC Huang Q Gillies J. T Reg–Specific Insulin Receptor Deletion Prevents Diet-Induced and Age-Associated Metabolic Syndrome. J Exp Med (2020) 217:e20191542. doi: 10.1084/jem.20191542 32478834 PMC7398165

[93] Sun W Dong H Balaz M Slyper M Drokhlyansky E Colleluori G. snRNA-Seq Reveals a Subpopulation of Adipocytes That Regulates Thermogenesis. Nature (2020) 587:98–102. doi: 10.1038/s41586-020-2856-x 33116305

[94] Virtue S Lelliott CJ Vidal-Puig A. What Is the Most Appropriate Covariate in ANCOVA When Analysing Metabolic Rate? Nat Metab (2021) 3:1585–5. doi: 10.1038/S42255-021-00505-5 34903886

[95] Müller TD Klingenspor M Tschöp MH. Revisiting Energy Expenditure: How to Correct Mouse Metabolic Rate for Body Mass. Nat Metab (2021) 3:1134–6. doi: 10.1038/S42255-021-00451-2 34489606

[96] Burýšek L Houštěk J. β-Adrenergic Stimulation of Interleukin-1α and Interleukin-6 Expression in Mouse Brown Adipocytes. FEBS Lett (1997) 411:83–6. doi: 10.1016/S0014-5793(97)00671-6 9247147

[97] Mauer J Chaurasia B Goldau J Vogt MC Ruud J Nguyen KD. Signaling by IL-6 Promotes Alternative Activation of Macrophages to Limit Endotoxemia and Obesity-Associated Resistance to Insulin. Nat Immunol (2014) 15:423–30. doi: 10.1038/ni.2865 PMC4161471 24681566

[98] Emanuelli B Vienberg SG Smyth G Cheng C Stanford KI Arumugam M. Interplay Between FGF21 and Insulin Action in the Liver Regulates Metabolism. J Clin Invest (2014) 124:515–27. doi: 10.1172/JCI67353 PMC3904602 24401271

[99] Hondares E Iglesias R Giralt A Gonzalez FJ Giralt M Mampel T. Thermogenic Activation Induces FGF21 Expression and Release in Brown Adipose Tissue. J Biol Chem (2011) 286:12983–90. doi: 10.1074/jbc.M110.215889 PMC3075644 21317437

[100] Chartoumpekis D v. Habeos IG Ziros PG Psyrogiannis AI Kyriazopoulou VE Papavassiliou AG. Brown Adipose Tissue Responds to Cold and Adrenergic Stimulation by Induction of FGF21. Mol Med (2011) 17:736. doi: 10.2119/MOLMED.2011.00075 21373720 PMC3146611

[101] Huang Z Zhong L Lee JTH Zhang J Wu D Geng L. The FGF21-CCL11 Axis Mediates Beiging of White Adipose Tissues by Coupling Sympathetic Nervous System to Type 2 Immunity. Cell Metab (2017) 26:493–508. doi: 10.1016/j.cmet.2017.08.003 28844880

[102] Rao RR Long JZ White JP Svensson KJ Lou J Lokurkar I. Meteorin-Like Is a Hormone That Regulates Immune-Adipose Interactions to Increase Beige Fat Thermogenesis. Cell (2014) 157:1279–91. doi: 10.1016/j.cell.2014.03.065 PMC4131287 24906147

[103] Cereijo R Gavaldà-Navarro A Cairó M Quesada-López T Villarroya J Morón-Ros S. CXCL14, a Brown Adipokine That Mediates Brown-Fat-To-Macrophage Communication in Thermogenic Adaptation. Cell Metab (2018) 28:750–63. doi: 10.1016/j.cmet.2018.07.015 30122557

[104] Campderrós L Moure R Cairó M Gavaldà-Navarro A Quesada-López T Cereijo R. Brown Adipocytes Secrete GDF15 in Response to Thermogenic Activation. Obesity (2019) 27:1606–16. doi: 10.1002/oby.22584 31411815

[105] Villarroya J Cereijo R Gavald -Navarro A Peyrou M Giralt M Villarroya F. New Insights Into the Secretory Functions of Brown Adipose Tissue. J Endocrinol (2019) 243:R19–27. doi: 10.1530/JOE-19-0295 31419785

[106] Scheele C Wolfrum C. Brown Adipose Crosstalk in Tissue Plasticity and Human Metabolism. Endocr Rev (2020) 41:53–65. doi: 10.1210/endrev/bnz007 PMC7006230 31638161

[107] Villarroya J Cereijo R Giralt M Villarroya F. Secretory Proteome of Brown Adipocytes in Response to cAMP-Mediated Thermogenic Activation. Front Physiol (2019) 10. doi: 10.3389/fphys.2019.00067 PMC6374321 30792664

[108] Deshmukh AS Peijs L Beaudry JL Jespersen NZ Nielsen CH Ma T. Proteomics-Based Comparative Mapping of the Secretomes of Human Brown and White Adipocytes Reveals EPDR1 as a Novel Batokine. Cell Metab (2019) 30:963–75. doi: 10.1016/j.cmet.2019.10.001 31668873

[109] Thomou T Mori MA Dreyfuss JM Konishi M Sakaguchi M Wolfrum C. Adipose-Derived Circulating miRNAs Regulate Gene Expression in Other Tissues. Nature (2017) 542:450–5. doi: 10.1038/nature21365 PMC5330251 28199304

[110] Chen Y Buyel JJ Hanssen MJW Siegel F Pan R Naumann J. Exosomal microRNA miR-92a Concentration in Serum Reflects Human Brown Fat Activity. Nat Commun (2016) 7:11420. doi: 10.1038/NCOMMS11420 27117818 PMC4853423

[111] Lynes MD Leiria LO Lundh M Bartelt A Shamsi F Huang TL. The Cold-Induced Lipokine 12,13-diHOME Promotes Fatty Acid Transport Into Brown Adipose Tissue. Nat Med (2017) 23:631–7. doi: 10.1038/NM.4297 PMC5699924 28346411

[112] Han MS White A Perry RJ Camporez JP Hidalgo J Shulman GI. Regulation of Adipose Tissue Inflammation by Interleukin 6. Proc Natl Acad Sci USA (2020) 117:2751–60. doi: 10.1073/pnas.1920004117 PMC7022151 31980524

[113] Martins FF Bargut TCL Aguila MB Mandarim-de-Lacerda CA. Thermogenesis, Fatty Acid Synthesis With Oxidation, and Inflammation in the Brown Adipose Tissue of Ob/Ob (–/–) Mice. Ann Anat (2017) 210:44–51. doi: 10.1016/j.aanat.2016.11.013 27986616

[114] Orava J Nuutila P Noponen T Parkkola R Viljanen T Enerbäck S. Blunted Metabolic Responses to Cold and Insulin Stimulation in Brown Adipose Tissue of Obese Humans. Obesity (2013) 21:2279–87. doi: 10.1002/oby.20456 23554353

[115] Okla M Zaher W Alfayez M Chung S. Inhibitory Effects of Toll-Like Receptor 4, NLRP3 Inflammasome, and Interleukin-1β on White Adipocyte Browning. Inflammation (2018) 41:626–42. doi: 10.1007/s10753-017-0718-y PMC6066287 29264745

[116] Shankar K Kumar D Gupta S Varshney S Rajan S Srivastava A. Role of Brown Adipose Tissue in Modulating Adipose Tissue Inflammation and Insulin Resistance in High-Fat Diet Fed Mice. Eur J Pharmacol (2019) 854:354–64. doi: 10.1016/j.ejphar.2019.02.044 30822393

